# Comparison of three-dimensional body centre of mass trajectories during locomotion through zero- and one-dimensional statistics

**DOI:** 10.1038/s41598-022-22635-w

**Published:** 2022-10-22

**Authors:** Francesco Luciano, Luca Ruggiero, Alberto Minetti, Gaspare Pavei

**Affiliations:** Locomotion Physiomechanics Laboratory–Division of Physiology, Department of Pathophysiology and Transplantation, University of Milan, Via Mangiagalli 32, 20133 Milan, Italy

**Keywords:** Physiology, Health care

## Abstract

The trajectory of the body centre of mass (BCoM) during locomotion differs through speeds, gaits and pathological states; statistical methods are needed to compare it among different conditions. Here, ten participants walked on treadmill at 1.1 and 1.4 m/s; BCoM trajectories were obtained through stereophotogrammetry and expressed as Fourier series. Trajectories were compared among speeds using (i) zero-dimensional (0D) linear and circular tests for difference on amplitudes and phases of Fourier series harmonic, (ii) one-dimensional statistical parametric mapping (1D-SPM) t-tests on the anteroposterior, mediolateral and vertical axial components of the BCoM trajectory and (iii) 1D-SPM Hotelling’s T^2^ test on the three-dimensional BCoM trajectory. Increasing speed increased the amplitude and decreased the phase of the fundamental (2nd) vertical harmonic. Coherently, the BCoM vertical trajectory featured greater displacement and a forward-shift, combined with greater displacement along the anteroposterior axis. Hotelling’s T^2^ 1D-SPM on the whole trajectory featured supra-threshold clusters at the transition between double and single support, and mid of the single support. 0D and 1D test yielded coherent and complementary results: 1D-SPM Hotelling’s T^2^ is suitable to compare whole three-dimensional BCoM trajectories; however, when researchers hypothesize that conditions may impact specific harmonics or axial components, 0D or 1D-SPM t-tests are recommended.

## Introduction

During terrestrial locomotion, the Body Centre of Mass (BCoM) moves along a complex three dimensional (3D) path^1–4^; characterizing such trajectory has applications both for basic science and translational medicine. The energy spent during locomotion largely depends on the work performed to raise and accelerate the BCoM^2,5–7^, and finely regulating its trajectory is a primary aim of motor control strategies^8^, especially when locomoting at high speeds, on irregular terrains^9^ and during gait turnings^10^. Hence, deviations from normal BCoM trajectories can be a marker of gait impairment in neurological and musculoskeletal diseases, obesity and aging^11–16^ and can guide diagnosis, treatment and rehabilitation of gait disorders^8,17,18^.

The 3D BCoM trajectory can be experimentally reconstructed by using force platforms or stereophotogrammetry. Force platforms record the ground reaction forces during gait; subsequent integrations with respect to time calculate BCoM speed and displacement^19^. Conversely, stereophotogrammetry allows to compute the position of the BCoM as the mass-weighted mean of all the body segments’ positions^20^.

Even though these procedures reconstruct the 3D BCoM trajectory during gait, researchers and clinicians still need methods to quantitatively compare such trajectory across different conditions. These methods are required to draw inferences on the mechanistic phenomena underlying changes in BCoM trajectories, and provide sensitive and specific information for patient management. The BCoM position is time-dependent, hence can be regarded as a one-dimensional quantity (1D) with respect to time. Representative points of the trajectory, such as its minimum and maximum value for each axial component, can be extracted and compared among conditions; this approach conducts time-independent (zero dimensional, 0D) statistical hypothesis tests, and may be an oversimplification when the null hypothesis pertains the whole trajectory^21,22^. On the other hand, reporting those trajectories as means surrounded by error clouds is appropriate for descriptive, but not for inferential purposes^23^. A less biased method would be to conduct a Fourier analysis, which expresses the cyclical variations of the BCoM position during strides as a sum of periodic functions—namely harmonics—each characterized by an amplitude and phase^4^. Harmonic amplitudes are defined through linear variables, so should be compared with ‘classical’ (linear) zero-dimensional tests, while harmonic phases are angular variables, so should be compared with zero-dimensional circular statistics^24,25^. Alternatively, the entire BCoM trajectory may be compared through Statistical Parametric Mapping, a 1D hypothesis test conducted on the whole 3D spatial trajectory or on its anteroposterior, mediolateral and vertical axial components^21,26^.

The aim of this study was to compare the BCoM trajectories during human walking at different speeds by using (i) linear and circular statistics on amplitude and phases, (ii) 1D SPM on the three separate anteroposterior, mediolateral and vertical BCoM trajectories, and (iii) 1D SPM on the 3D BCoM trajectory. Previous literature showed that the higher the walking speed, the higher the displacement of the BCoM on the vertical axis and the lower the mediolateral displacement^4,27^; this impacts on walking mechanics and energetics^16,28^, stability and risk of falling^8,29^. Characterising speed-dependent variations in the BCoM trajectory is needed in clinical gait analysis; indeed, some gait pathologies can lead to excessive BCoM displacement, but their impact on BCoM trajectory may be masked by the speed-dependent variations of the normal trajectory itself^29^. Finally, by comparing 0D and 1D methods, the present investigation is meant to propose the proper usage for each one when assessing differences in BCoM trajectories.

## Materials and methods

**Figure 1:**
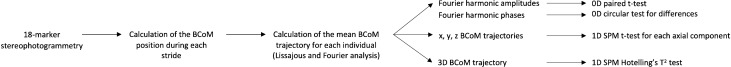
Data analysis and statistics flowchart. BCoM: body centre of mass. 3D: three-dimensional. 0D: Zero-dimensional. 1D: one-dimensional.

The BCoM trajectories of 10 participants walking on treadmill at 1.1 and 1.4 m/s were compared in a repeated-measure protocol. Firstly, preliminary data were collected to conduct sample size estimation; subsequently, an independent sample of participants was recruited. The BCoM trajectory was reconstructed through stereophotogrammetry with reflective markers, Lissajous contour and Fourier analysis. Statistical testing for speed-dependent differences was conducted using (i) 0D linear t-tests for amplitudes of Fourier harmonics, and circular tests for difference in phases (ii) 1D-SPM t-tests on the three axial BCoM trajectories, and (iii) 1D-SPM Hotelling’s T^2^ test on the 3D trajectory (see Fig. 1 for study flowchart).

### Sample size estimation

A priori SPM power analysis was conducted. A sample size of 10 participants was hence selected to keep false-positive and false-negative error below 0.05 and 0.10, respectively, while comparing axial trajectories with SPM-t-test^30–32^. Further information is provided in Supplementary Materials. Analyses were conducted using Python 2.7.15, Numpy, Pandas and Power1D^30,33,34^.

### Participants

Ten healthy participants (5 females, 5 males; age 24 ± 4 years; height 1.69 ± 0.06 m; mass 60 ± 9 kg; mean ± SD) were recruited; participants were excluded if they had any neurological, musculoskeletal or joint disorder, or any other condition that could impact on gait. Informed consent was obtained from all participants. All procedures were approved by the local Ethical Committee and complied with the Declaration of Helsinki. All experiments were performed in accordance with relevant guidelines and regulations.

### Kinematics and Fourier analysis

Participants walked on a motorised treadmill (Ergo LG, Woodway) at 1.1 and 1.4 m/s. An eight-camera system (Vicon MX, Oxford Metrics) measured the spatial coordinates of 18 reflective markers to construct an 11-segment model of the human body, as described by Pavei and colleagues^20^. For each segment, standard tables of Dempster^35^ defined its mass as a fraction of total body mass, and the position of its centre of mass with respect to the proximal joint. Acquisitions had a sampling rate of 100 Hz and lasted 1 min. The average 3D BCoM position was calculated according to the procedures presented by Minetti and colleagues^4^. Briefly, individual strides were extracted by trimming the BCoM trajectory every two vertical maxima; each stride was then interpolated to 201 points between 0 and 2π. For each component (anteroposterior: x; mediolateral: y; vertical: z), the BCoM position at the end of each stride was forced to correspond to the position at the start of the stride by progressively filling the gap along the cycle (i.e., to close the loop and form a continuous 3D Lissajous contour^4^). The 3D BCoM trajectory for each stride was then defined by the amplitudes and phases of a 10-harmonic Fourier series, aligned to start with the closest zero-crossing of the mediolateral component from right to left, as in Minetti et al.^4^, in the form:

$$\widehat{x}\left(t\right)=\sum_{i=1}^{10}{a}_{i}^{x}\mathrm{sin}(it+{\phi }_{i}^{x})$$

$$\widehat{y}\left(t\right)=\sum_{i=1}^{10}{a}_{i}^{y}\mathrm{sin}(it+{\phi }_{i}^{y})$$

$$\widehat{z}\left(t\right)=\sum_{i=1}^{10}{a}_{i}^{z}\mathrm{sin}(it+{\phi }_{i}^{z})$$

where *a*_*i*_ and *ϕ*_*i*_ are the amplitude and phase of the *i*th harmonic for a given component (see Ref.^4^ for details). For each component, harmonic (1st to 10th), and speed, individual amplitudes and phases were averaged with linear and circular statistics, respectively^36^; this allowed to describe each individual’s average BCoM trajectory^4^. Only mean amplitudes and phases, and their SDs, were used for 0D hypothesis testing while the representative mean BCoM trajectories, reconstructed as a sum of harmonics for each component, were considered for 1D hypothesis testing using SPM.

### Zero-dimensional hypothesis testing on Fourier harmonics

Differences between harmonics amplitudes at 1.1 vs 1.4 m/s were tested using a paired t-test. For each amplitude *a*_*i*_ of each ith harmonics on each anteroposterior, mediolateral and vertical axis, the null and alternative hypotheses were:

$$H0{:}\; {a}_{{i}_{1.1 m/s}}={a}_{{i}_{1.4 m/s}}$$

$$H1{:}\; {a}_{{i}_{1.1 m/s}}\ne {a}_{{i}_{1.4 m/s}}$$

Differences between harmonic phases at 1.1 vs 1.4 m/s were tested using a circular paired test for differences. Firstly, each pairwise difference in harmonic phase was checked for circular uniformity using the Rayleigh test^36^; as the p-value was inferior to 0.027 for all measures, unimodal deviations from circular uniformity of the residuals were confirmed. In all cases, the distribution of the pairwise difference of each harmonic phase was modelled with a von Mises probability function; estimated concentration parameters (κ) were between 1.2 and 19.7. For each phase *ϕ*_*i*_ of each ith harmonic on each axis, the null and alternative hypotheses were:

$$H0{:}\; {\phi }_{{i}_{1.1 m/s}}={\phi }_{{i}_{1.4 m/s}}$$

$$H1{:}\; {\phi }_{{i}_{1.1 m/s}}\ne {\phi }_{{i}_{1.4 m/s}}$$

In order to control for the overall false-positive statistical error, a Benjamini–Hochberg correction for multiple comparisons was implemented^37^. Statistical analyses were done with Matlab R2021a (MathWorks, Natick, MA), R 3.6.2 and R Studio^38^.

### One-dimensional SPM hypothesis testing on the separate axial BCoM trajectories

Given ***μ***_*x*(t)_ the mean BCoM trajectory time series for the anteroposterior axis at 1.1 and 1.4 m/s, the null and alternative hypotheses were:

$$H0{:}\; {{\varvec{\mu}}}_{{x(t)}_{1.1 m/s}}={{\varvec{\mu}}}_{{x(t)}_{1.4 m/s}}$$

$$H1{:}\; {{\varvec{\mu}}}_{{x(t)}_{1.1 m/s}}\ne {{\varvec{\mu}}}_{{x(t)}_{1.4 m/s}}$$

Student’s SPM{t} test statistics and critical SPM{t}* were computed given a false-positive error level of 0.05. The null hypothesis was rejected if SPM{t} statistics exceeded the critical SPM{t}* at least once during stride. The same procedure was repeated for comparisons on the mediolateral and vertical axes.

### One-dimensional SPM hypothesis testing on the 3D BCoM trajectory

Given ***μ***_(t)_ the mean three-dimensional BCoM trajectory time series at 1.1 and 1.4 m/s, the null and alternative hypotheses were:

$$H0{:}\; {{\varvec{\mu}}}_{{(t)}_{1.1 m/s}}={{\varvec{\mu}}}_{{(t)}_{1.4 m/s}}$$

$$H1{:}\; {{\varvec{\mu}}}_{{(t)}_{1.1 m/s}}\ne {{\varvec{\mu}}}_{{(t)}_{1.4 m/s}}$$

Hotelling’s SPM{T^2^} test statistics and critical SPM{T^2^}* were computed given a false-positive error probability of 0.05. The null hypothesis was rejected if SPM{T^2^} statistics exceeded the critical SPM{T^2^}* at least once during the stride. Waveform registration^26^ had already been achieved by aligning all the waveforms so that the phase of the fundamental mediolateral (y) harmonics was set to zero for all acquisitions (see “Kinematics and Fourier analysis” section). Analyses were done using Python 2.7.15, Numpy, Pandas and Power1D^30,33,34^.

## Results

**Figure 2:**
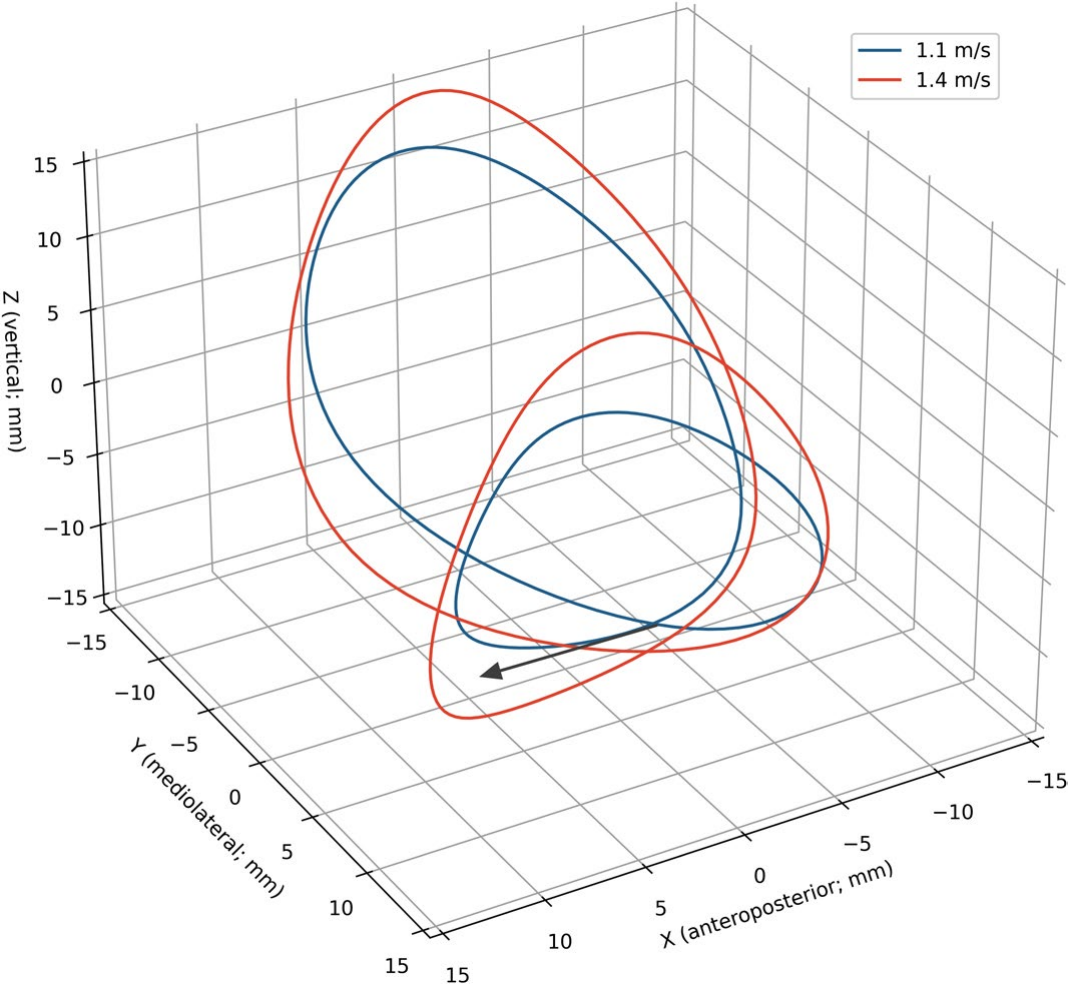
Mean three-dimensional trajectory of the BCoM at 1.1 and 1.4 m/s. The arrow indicates the walking progression direction. The trajectories form a closed loop since participants were walking on a treadmill; equivalent closed loops would have been obtained from ground walking by subtracting the displacement due to the average forward progression speed.

Mean three-dimensional trajectories of the BCoM when walking at 1.1 and 1.4 m/s are shown in Fig. 2.

### Zero-dimensional hypothesis testing on Fourier harmonics

**Figure 3:**
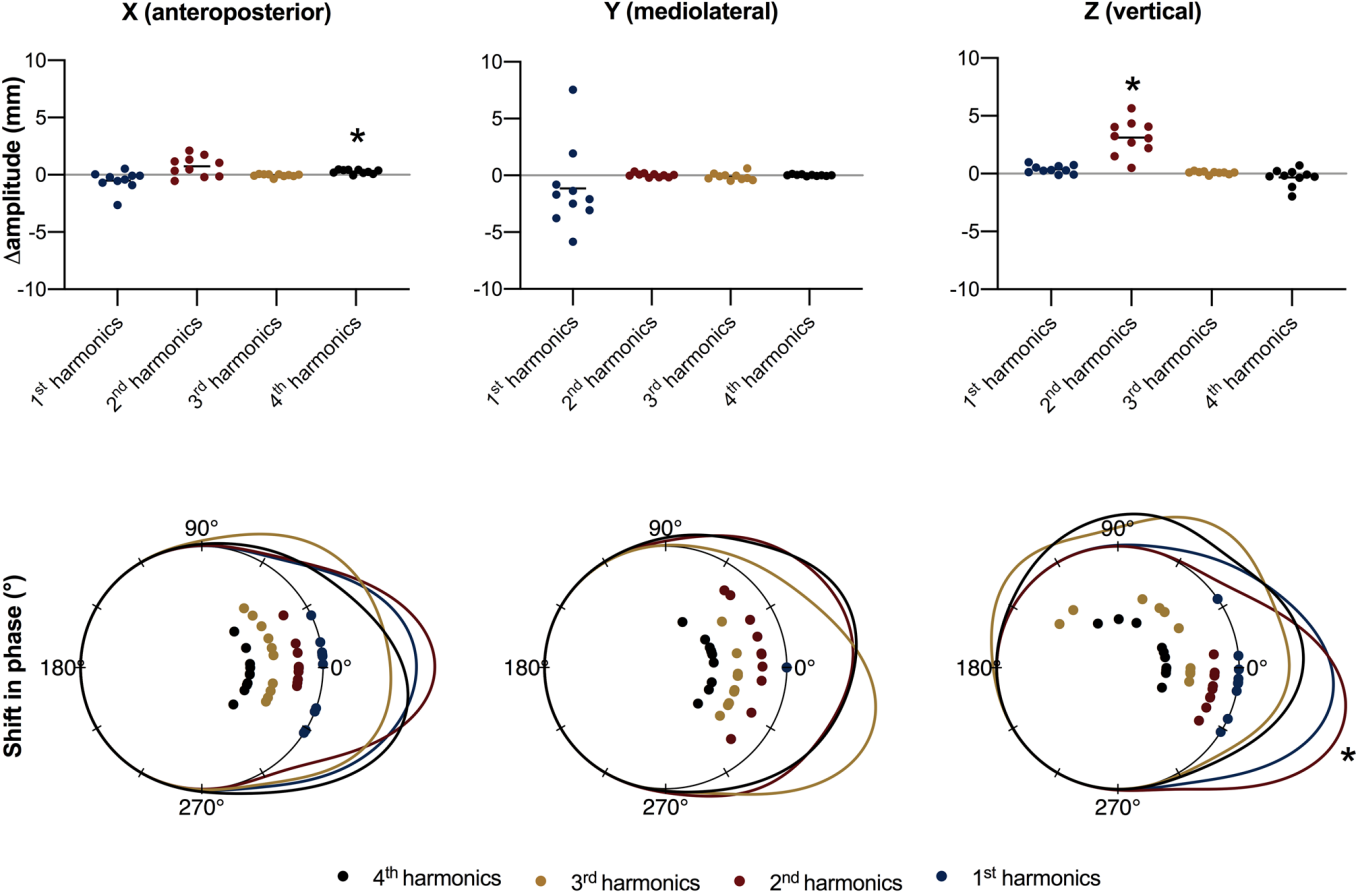
Variations in amplitudes and phases of the first four Fourier harmonics for each axis, while walking at 1.4 m/s as compared with 1.1 m/s. Top panel: Individual speed-dependent variations in harmonics amplitudes. Bottom panel: Individual speed-dependent variations in harmonic phases. Probability density functions for circular variables are shown as continuous curves. In Fourier analysis, the phase of the first mediolateral (y) harmonic was conventionally set to zero for all trajectories in order to align them. *p < 0.05; paired linear or circular test for differences with Benjamini–Hochberg correction for multiple comparisons.

Figure 3 depicts within-subject variations in amplitudes and phases of the first 4 harmonics for each axial component, when walking at 1.4 m/s versus 1.1 m/s. With greater speed, the amplitude of the 4th anteroposterior harmonic increased, while the amplitude of the 2nd (fundamental) vertical harmonic increased and its phase decreased (i.e., the trajectory shifted forward). Table S1 of the Supplementary Material reports mean amplitudes, standard deviations, mean phases and radii for all harmonics at 1.1 and 1.4 m/s, while Table S2 shows extended results for the linear and circular statistical tests.

### One-dimensional SPM hypothesis testing on the separate axial BCoM trajectories

**Figure 4:**
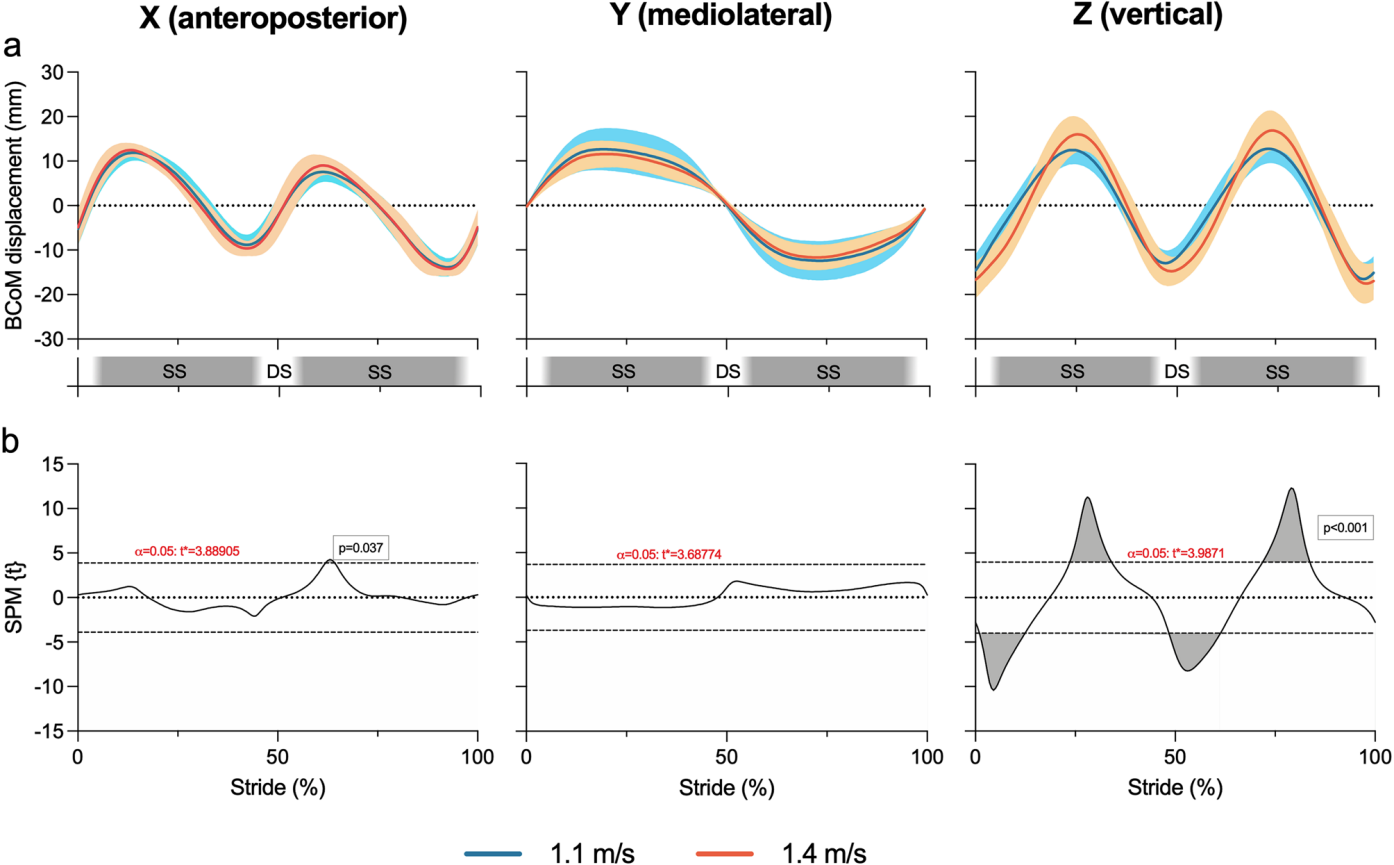
Anteroposterior (x), mediolateral (y) and vertical (z) trajectories of the BCoM at 1.1 and 1.4 m/s (**a**) and corresponding SPM paired two-tailed t-test with an alpha level of 0.05 (**b**). For graphical purposes, the horizontal axis spans from 0 to 100% of the stride period, instead of spanning from 0 to 2π as required by Fourier analysis. In panel a, approximate double support (DS) and single support (SS) periods are shown to ease the understanding of the plots.

The anteroposterior, mediolateral, and vertical BCoM trajectories at 1.1 versus 1.4 m/s are depicted in Fig. 4a. When comparing such trajectories through the SPM paired t-tests, the critical SPM{t} threshold was exceeded on the anteroposterior (x) and vertical (z) axes; hence, the null hypothesis was rejected in these two cases (Fig. 4b; p < 0.05). The largest differences were observed on the vertical axis, where with greater speed the BCoM displacement increased and the BCoM trajectory shifted forward (Fig. 4a).

### One-dimensional SPM hypothesis testing on the 3D BCoM trajectory

**Figure 5:**
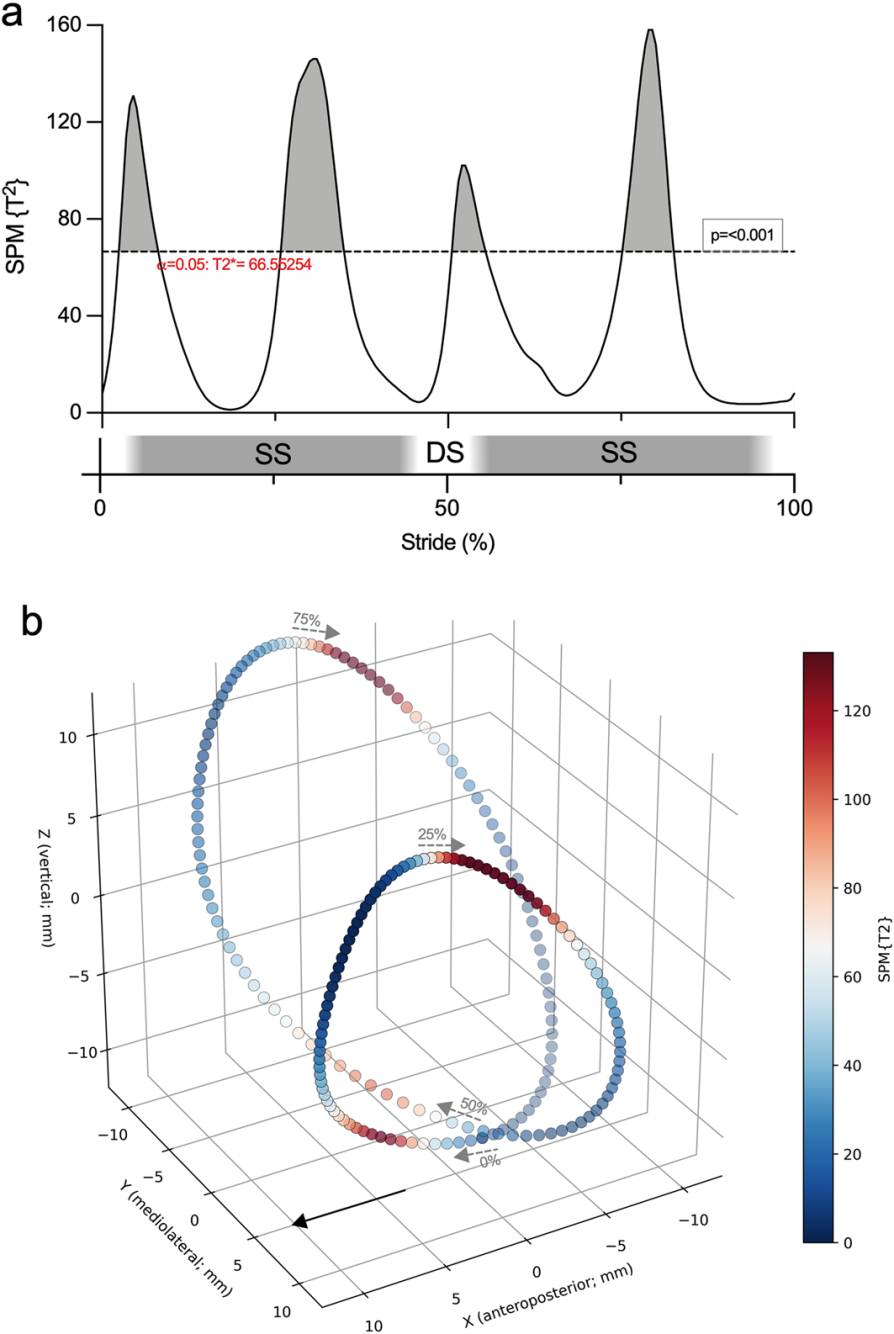
SPM paired Hotelling’s T^2^ test on the three-dimensional BCoM trajectory while walking at 1.1 m/s versus 1.4 m/s. Approximate double support (DS) and single support (SS) periods are shown to ease the understanding of the plot (**a**). The same results are mapped, by referring to the illustrated color ramp on the right, on each point of the mean 3D BCoM trajectory at 1.1 m/s (**b**). The greater the SPM{T^2^}, the greater the differences between the two compared conditions. The black, solid arrow indicates the walking progression direction; 0%, 25%, 50% and 75% of gait cycle are indicated together with the local BCoM displacement direction (dashed grey arrows).

Figure 5a shows the Hotelling’s SPM{T^2^} statistics and critical threshold for the comparison of the 3D BCoM trajectories at 1.1 versus 1.4 m/s; the SPM{T^2^} statistics is also color-mapped on the BCoM contour at 1.1 m/s in Fig. 5b. At the transition between double support and single support, the BCoM at 1.4 m/s was more caudal than at 1.1 m/s, while in the mid of the single support the BCoM at 1.4 m/s was more medial and cranial than at 1.1 m/s (Figs. 2, 5).

## Discussion

Increasing walking speed incremented the vertical and anteroposterior BCoM excursion, and shifted forward the vertical component. Zero-dimensional and 1D statistical tests yielded coherent but complementary results: while the former showed amplitude and phase differences in the vertical fundamental harmonic, the latter confirmed such results and highlighted where in the cycle divergences mostly occurred.

### Zero-dimensional hypothesis testing on Fourier harmonics

Fourier analysis expressed the cyclical trajectory as a sum of harmonics, each characterized by an amplitude and phase; increasing speed increased the amplitude of the 2nd (or ‘fundamental’) vertical harmonic and decreased its phase, indicating greater excursion of the BCoM and a forward shift in its trajectory (Fig. 3). It was already known that the higher the walking speed, the greater the vertical displacement of BCoM^4,27,29^; besides differences in amplitudes, the present findings strongly encourage to assess shifts in phase, as previously suggested by Minetti et al. and Takiyama et al.^4,27^.

While distinctly comparing amplitudes and phases of single harmonics can unveil fine differences in gait, this method faces the problem of multiple statistical comparisons: the greater the number of compared harmonics, the greater the number of independent statistical tests, the higher the overall false-positive error. In the present paper, Benjamini–Hochberg correction for multiplicity was chosen to control for false-discoveries, though this reduces statistical power^37^. Zero-dimensional testing on Fourier harmonics can hence be the method of choice when researchers have a priori knowledge on the mechanistic or diagnostic role of changes in specific harmonics—thus restricting the number of planned statistical comparisons—or as an exploratory analysis after 1D tests. Of note, the quantitative description of the BCoM trajectory through Fourier harmonics and Lissajous contours has great value even when no 0D statistical tests are further applied, as it always allows to determine the mean BCoM trajectory over several strides within and between participants.

### One-dimensional SPM hypothesis testing on the separate axial BCoM trajectories

One-dimensional SPM t-test found four supra-threshold clusters for the vertical component of the BCoM trajectory, coherently with 0D statistics. Clusters were symmetrical, highlighting that an increase in speed similarly affected the first and the second half of the stride (Fig. 4). The changes in the vertical trajectory of BCoM were similar to the ones described by Takiyama et al.^27^. However, contrary to previous evidence, no significant variation in mediolateral sway was found, potentially because the speeds were close or there was a dispersion in data (Fig. 4a). Moreover, a small supra-threshold cluster was highlighted in the anteroposterior axial comparison.

Testing hypotheses on specific axial components of the BCoM trajectory can be the best choice when the movement on one axis has a precise physiological or clinical meaning. For instance—all other parameters being equal—the higher the displacement of the BCoM along the vertical axis, the higher the mechanical work required in locomotion; the vertical trajectory of BCoM is also pivotally linked with the energy-saving mechanisms that can reduce the cost of transport^2,5,7,16,39^. Moreover, the ability to control the mediolateral sway of the BCoM can be altered in aging and clinical gait impairments in a speed-dependent way; in such circumstances, the altered mediolateral displacement of the BCoM increases the risk of sideways falling^8,12^. In all these examples, precise hypothesis can be drawn along individual axial components.

### One-dimensional SPM hypothesis testing on the 3D BCoM trajectory

One-dimensional SPM Hotelling’s T^2^ test found similar results to the three independent SPM t-test on axial components. When increasing walking speed from 1.1 to 1.4 m/s, the BCoM was more caudal at the transition between double support and single support, and more cranial during the mid of the single support (Figs. 4 and 5); neither 0D tests nor 1D SPM on the separate axial BCoM trajectories highlighted differences in the mediolateral component between speeds, where only small differences were observed through descriptive statistics (Figs. 3 and 4).

Even though the two SPM analyses yielded coherent results, separately testing the three components de facto assumes them as independent, while conducting a unique SPM T^2^ test on the whole BCoM trajectory takes into account concurrent variations along the axial components. The latter is undoubtedly useful in walking, when potential energy (dependent on the vertical BCoM position) and kinetic energy (mostly dependent on the anteroposterior BCoM velocity) exchange in a pendulum-like manner^2^, which makes the axial trajectories dependent on each other. Thus, SPM Hotelling’s T^2^ test is suitable when the tested hypothesis pertains the whole trajectory, and may be followed by post-hoc SPM-t-tests on axial components^40^. As the 3D BCoM trajectory during ambulation constitutes a “locomotor signature” for humans and terrestrial animals^4^ and varies at different speeds, gaits, physiological and pathological conditions^4,8,11–14,27,41^, a comprehensive test on its shape can be a specific and/or sensitive marker of disease progression, recovery or response to therapy. Moreover, the BCoM trajectory undergoes fine redirection in the three dimensions while turning gaits and negotiating obstacles^9,10^; in such case, studying it under a spatial rather than axial point of view may be more powerful in unveiling the motor control strategies that humans and animals implement to face deviations in gait.

SPM analysis of 2- or 3-D trajectories requires *landmark definition* (i.e., definition of homologous points among trajectories) and *spatial registration* (i.e., translation, rotation or scaling of trajectories in order to align landmarks^26^). Both steps are straightforward when analysing 3D BCoM trajectories, and were hereby obtained by setting the phase of the first mediolateral Fourier harmonic to zero for all the trajectories, hence ensuring that they all started at the inversion of the BCoM mediolateral sway from right to left^4^. Alternatively, trajectories can be aligned based on other gait events identified through kinematics, although considerable variability may be introduced depending on the chosen algorithm for the determination of stance and swing^42^.

## Future perspectives

The current investigation compared BCoM trajectories of healthy participants while walking at 1.1 and 1.4 m/s—two close speeds—in order to test the ability of the present statistical methods to detect subtle differences. The proposed framework of analysis of the BCoM trajectory can then be used to assess differences in motion among further physiological and pathological conditions. Researchers’ hypotheses may pertain not only a certain harmonic or axial trajectory, but also a specific region of the BCoM trajectory which corresponds to a gait event. In this case, Region of Interest (ROI) hypothesis testing can be implemented in SPM analysis^43^.

## Conclusions

The 3D BCoM trajectory during locomotion can be compared through zero-dimensional methods based on Fourier analysis, and one-dimensional SPM. Hotelling’s T^2^ SPM test compares whole three-dimensional trajectories among different conditions, compositely testing the variations along anteroposterior, mediolateral and vertical axes; such test is suitable when no specific hypotheses are drawn pertaining a single axis or harmonic. Conversely, researchers may hypothesise that a certain condition impacts on specific harmonics or axial components; in this case, they can implement SPM on single components, or zero-dimensional statistics on amplitudes and phases of Fourier harmonics.

## Supplementary Information

Supplementary Information.

## Data Availability

The datasets collected and/or analyzed during the current study are available from the corresponding author upon request.
