# Supplementary material for: Comparison of three-dimensional body centre of mass trajectories during locomotion through zero- and one-dimensional statistics
Source: Sci Rep. 2022 Oct 22;12:17777. doi: 10.1038/s41598-022-22635-w (PMC9588033; doi:10.1038/s41598-022-22635-w)
Supplement: Supplementary file 1 — Supplementary Information. [file 41598_2022_22635_MOESM1_ESM.docx]

**Supplementary Material**

**2. Materials and methods**

*Sample size estimation.* The sample size was estimated using *a priori* statistical parametric mapping (SPM) power analysis [S1]. Briefly, this procedure requires to define a null (H0) and an alternative (H1) effect, make assumptions on the amplitude and smoothness of the noise of the experimental data, and select a false-positive and false-negative statistical error level [S1–S3]. The null and alternative effects were defined based on Minetti et al., [S4], which provided exploratory data on speed-dependent differences in BCoM trajectory during walking. The trajectory of the BCoM during walking at 1.1 and 1.4 m/s on the mediolateral (y) and vertical (z) axes was reconstructed from the published amplitudes and phases of Fourier harmonics as:

$$\hat{y}\left( t \right)=\sum_{i=1}^{6} c_{i}^{y}sin(it+\phi_{i}^{y})$$

$$\hat{z}\left( t \right)=\sum_{i=1}^{6} c_{i}^{z}sin(it+\phi_{i}^{z})$$

where *c_i_* and *φ_i_* are the amplitude and phase of the i^th^ harmonic of the Fourier series, respectively. Hence, reconstructed curves served as experimentally observed effects for the SPM on the mediolateral and vertical axes. These two BCoM axial components were selected since previous literature showed they are the most impacted by speed [S5]. Noise amplitude (alternatively regarded as noise Sigma [S1]) and smoothness were calculated from exploratory unpublished data on 10 participants recruited independently from the actual study sample. This yielded an estimated noise amplitude (NAMP) of 3.9 mm and smoothness (NFWHM) of 20% for paired comparisons on the y axis, and NAMP = 1.4 and NFWHM = 20% on the z axis. A required sample size of 10 participants was hence estimated for a paired study design comparing trajectories using a 1D SPM t-test for each axial component, keeping the type I error below 0.05 and the type II error below 0.10 (i.e., achieving a statistical power of 0.9).


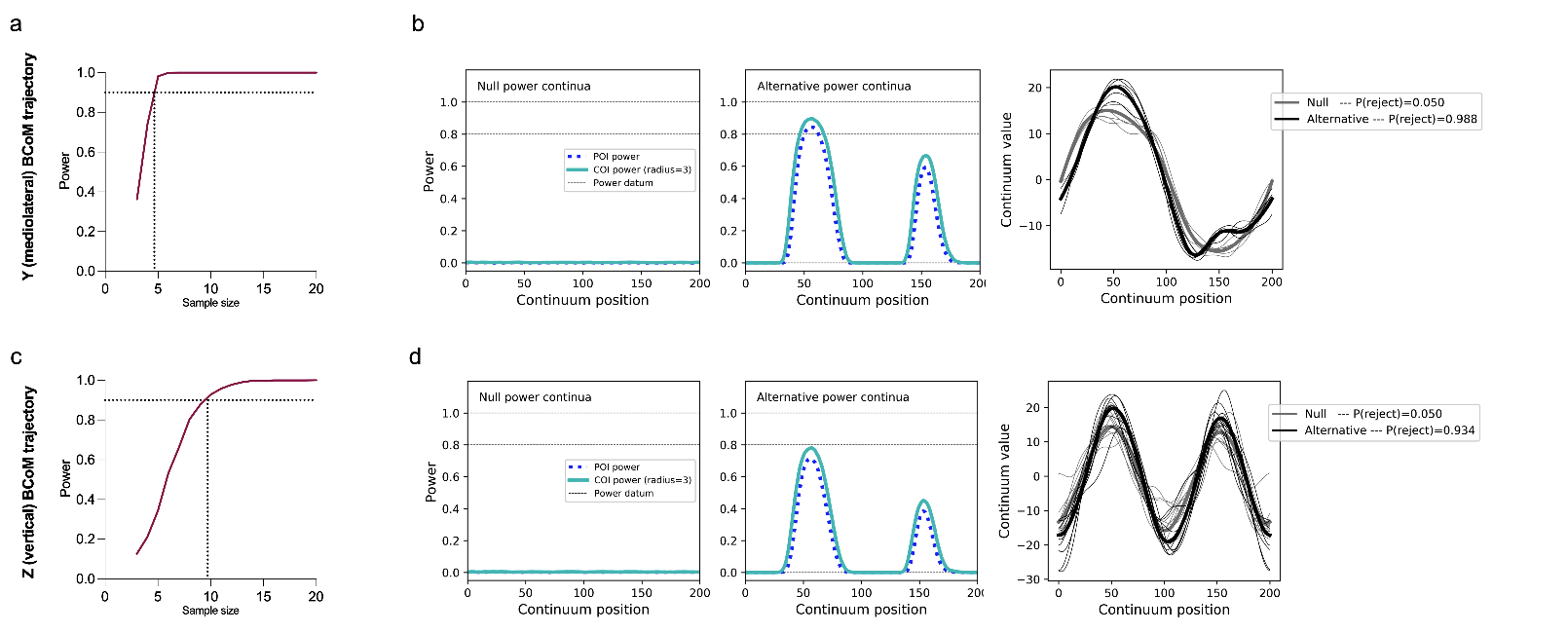


*Figure S1*. *A priori* Statistical Parametric Mapping (SPM) power analysis for the mediolateral (a, b) and vertical (c, d) components of the Body Centre of Mass (BCoM) trajectories. Experimental data from Minetti et al. [S4] were used to estimate 1D signal characteristics, and exploratory data on 10 participants (independent from the study sample) were used to assess noise characteristics. The relations between omnibus statistical power and sample size for the hypothesis testing on the vertical and lateral BCoM trajectory components are shown in panels a and c, respectively. Panels b and d show the modelled null and alternative effects and report the power analysis results for the point-of interest (POI) and center-of interest (COI) continuum. See Pataky [S1] for details on such variables.

**3. Results**

*Table S1.* Mean amplitudes, standard deviations, mean phases and radii for all harmonics of the Fourier series along the anteroposterior, mediolateral, and vertical axial components of the Body Centre of Mass trajectory, at 1.1 and 1.4 m/s.

|  | | | **Linear variables** | |  |  | **Circular variables** | |
| --- | --- | --- | --- | --- | --- | --- | --- | --- |
| **Speed (m/s)** | **Axial component** |  | **Mean (mm)** | **Standard deviation (mm)** |  |  | **Mean (rad)** | **Radius** |
| 1.1 | Anteroposterior (x) | Amplitude 1 | 3.76 | 1.37 |  | Phase 1 | -0.18 | 0.94 |
|  |  | Amplitude 2 | 10.76 | 1.61 |  | Phase 2 | -0.35 | 0.97 |
|  |  | Amplitude 3 | 0.51 | 0.14 |  | Phase 3 | -0.89 | 0.84 |
|  |  | Amplitude 4 | 1.88 | 0.38 |  | Phase 4 | -0.06 | 0.92 |
|  |  | Amplitude 5 | 0.31 | 0.09 |  | Phase 5 | -0.76 | 0.78 |
|  |  | Amplitude 6 | 0.41 | 0.12 |  | Phase 6 | -0.29 | 0.87 |
|  |  | Amplitude 7 | 0.12 | 0.03 |  | Phase 7 | -0.32 | 0.89 |
|  |  | Amplitude 8 | 0.18 | 0.08 |  | Phase 8 | -0.33 | 0.74 |
|  |  | Amplitude 9 | 0.07 | 0.02 |  | Phase 9 | -0.07 | 0.86 |
|  |  | Amplitude 10 | 0.09 | 0.05 |  | Phase 10 | -0.04 | 0.84 |
|  | Mediolateral (y) | Amplitude 1 | 13.40 | 5.15 |  | Phase 1 | 0.00 | 1.00 |
|  |  | Amplitude 2 | 0.61 | 0.16 |  | Phase 2 | 0.06 | 0.70 |
|  |  | Amplitude 3 | 1.45 | 0.68 |  | Phase 3 | -0.54 | 0.96 |
|  |  | Amplitude 4 | 0.18 | 0.05 |  | Phase 4 | -0.55 | 0.87 |
|  |  | Amplitude 5 | 0.22 | 0.08 |  | Phase 5 | -0.73 | 0.89 |
|  |  | Amplitude 6 | 0.08 | 0.03 |  | Phase 6 | 0.07 | 0.92 |
|  |  | Amplitude 7 | 0.07 | 0.02 |  | Phase 7 | -0.02 | 0.80 |
|  |  | Amplitude 8 | 0.05 | 0.02 |  | Phase 8 | 0.07 | 0.91 |
|  |  | Amplitude 9 | 0.06 | 0.03 |  | Phase 9 | -0.36 | 0.75 |
|  |  | Amplitude 10 | 0.03 | 0.01 |  | Phase 10 | 0.35 | 0.93 |
|  | Vertical (z) | Amplitude 1 | 2.08 | 0.85 |  | Phase 1 | -1.05 | 0.56 |
|  |  | Amplitude 2 | 13.31 | 2.82 |  | Phase 2 | -1.38 | 0.99 |
|  |  | Amplitude 3 | 0.73 | 0.21 |  | Phase 3 | -0.28 | 0.62 |
|  |  | Amplitude 4 | 1.38 | 0.65 |  | Phase 4 | -0.30 | 0.86 |
|  |  | Amplitude 5 | 0.25 | 0.05 |  | Phase 5 | -0.75 | 0.86 |
|  |  | Amplitude 6 | 0.46 | 0.13 |  | Phase 6 | -0.51 | 0.90 |
|  |  | Amplitude 7 | 0.10 | 0.02 |  | Phase 7 | -0.42 | 0.90 |
|  |  | Amplitude 8 | 0.21 | 0.07 |  | Phase 8 | -0.49 | 0.79 |
|  |  | Amplitude 9 | 0.05 | 0.01 |  | Phase 9 | -0.30 | 0.91 |
|  |  | Amplitude 10 | 0.08 | 0.04 |  | Phase 10 | -0.26 | 0.73 |
|  | | | | |  |  | | |
| 1.4 | Anteroposterior (x) | Amplitude 1 | 3.27 | 0.76 |  | Phase 1 | -0.27 | 0.93 |
|  |  | Amplitude 2 | 11.50 | 1.49 |  | Phase 2 | -0.30 | 0.96 |
|  |  | Amplitude 3 | 0.47 | 0.12 |  | Phase 3 | -0.71 | 0.84 |
|  |  | Amplitude 4 | 2.16 | 0.48 |  | Phase 4 | -0.16 | 0.89 |
|  |  | Amplitude 5 | 0.32 | 0.11 |  | Phase 5 | -0.29 | 0.58 |
|  |  | Amplitude 6 | 0.34 | 0.10 |  | Phase 6 | -0.39 | 0.93 |
|  |  | Amplitude 7 | 0.13 | 0.04 |  | Phase 7 | -0.43 | 0.84 |
|  |  | Amplitude 8 | 0.16 | 0.09 |  | Phase 8 | -0.07 | 0.88 |
|  |  | Amplitude 9 | 0.07 | 0.03 |  | Phase 9 | 0.01 | 0.91 |
|  |  | Amplitude 10 | 0.09 | 0.04 |  | Phase 10 | 0.16 | 0.85 |
|  | Mediolateral (y) | Amplitude 1 | 12.24 | 3.27 |  | Phase 1 | 0.00 | 1.00 |
|  |  | Amplitude 2 | 0.64 | 0.23 |  | Phase 2 | 0.24 | 0.58 |
|  |  | Amplitude 3 | 1.35 | 0.59 |  | Phase 3 | -0.72 | 0.89 |
|  |  | Amplitude 4 | 0.19 | 0.08 |  | Phase 4 | -0.40 | 0.73 |
|  |  | Amplitude 5 | 0.23 | 0.10 |  | Phase 5 | -0.63 | 0.95 |
|  |  | Amplitude 6 | 0.08 | 0.03 |  | Phase 6 | -0.22 | 0.83 |
|  |  | Amplitude 7 | 0.07 | 0.02 |  | Phase 7 | -0.03 | 0.90 |
|  |  | Amplitude 8 | 0.05 | 0.02 |  | Phase 8 | 0.10 | 0.87 |
|  |  | Amplitude 9 | 0.05 | 0.02 |  | Phase 9 | -0.16 | 0.80 |
|  |  | Amplitude 10 | 0.03 | 0.01 |  | Phase 10 | 0.17 | 0.89 |
|  | Vertical (z) | Amplitude 1 | 2.45 | 1.05 |  | Phase 1 | -1.31 | 0.53 |
|  |  | Amplitude 2 | 16.45 | 3.59 |  | Phase 2 | -1.59 | 0.98 |
|  |  | Amplitude 3 | 0.82 | 0.22 |  | Phase 3 | 0.59 | 0.83 |
|  |  | Amplitude 4 | 1.08 | 0.58 |  | Phase 4 | 0.23 | 0.60 |
|  |  | Amplitude 5 | 0.20 | 0.06 |  | Phase 5 | -0.29 | 0.66 |
|  |  | Amplitude 6 | 0.38 | 0.13 |  | Phase 6 | -0.14 | 0.72 |
|  |  | Amplitude 7 | 0.11 | 0.04 |  | Phase 7 | -0.30 | 0.84 |
|  |  | Amplitude 8 | 0.18 | 0.08 |  | Phase 8 | -0.20 | 0.91 |
|  |  | Amplitude 9 | 0.05 | 0.02 |  | Phase 9 | -0.17 | 0.88 |
|  |  | Amplitude 10 | 0.08 | 0.04 |  | Phase 10 | 0.07 | 0.90 |

*Table S2.* Speed-dependent differences in amplitudes and phases of the Fourier harmonics. For each harmonic, amplitudes were compared through a linear paired 0D t-test and phases through a circular paired 0D test for difference in means, with a type I (false-positive) error level of 0.05. The table reports p-values both uncorrected and corrected for multiple comparisons with Benjamini- Hochberg method. During Fourier analysis, the phase of the first mediolateral (y) harmonics was purposely set to zero for all trajectories in order to align them; hence, no statistical comparison was conducted on such variable.

|  |  | **Linear paired t-test** | |  |  | **Circular paired test for difference in means** | |
| --- | --- | --- | --- | --- | --- | --- | --- |
|  |  | **Uncorrected p-value** | **Corrected p-value** |  |  | **Uncorrected p-value** | **Corrected p-value** |
| Anteroposterior (x) | Amplitude 1 | 0.102 | 0.338 |  | Phase 1 | 0.527 | 0.695 |
|  | Amplitude 2 | **0.027** | 0.153 |  | Phase 2 | 0.519 | 0.695 |
|  | Amplitude 3 | 0.343 | 0.616 |  | Phase 3 | 0.431 | 0.679 |
|  | Amplitude 4 | **0.001** | **0.010** |  | Phase 4 | 0.326 | 0.654 |
|  | Amplitude 5 | 0.632 | 0.757 |  | Phase 5 | **0.038** | 0.203 |
|  | Amplitude 6 | 0.130 | 0.390 |  | Phase 6 | 0.350 | 0.654 |
|  | Amplitude 7 | 0.355 | 0.616 |  | Phase 7 | 0.681 | 0.825 |
|  | Amplitude 8 | 0.390 | 0.616 |  | Phase 8 | 0.891 | 0.923 |
|  | Amplitude 9 | 0.931 | 0.931 |  | Phase 9 | 0.687 | 0.825 |
|  | Amplitude 10 | 0.155 | 0.422 |  | Phase 10 | 0.063 | 0.203 |
| Mediolateral (y) | Amplitude 1 | 0.345 | 0.616 |  | Phase 1 | NA | NA |
|  | Amplitude 2 | 0.663 | 0.757 |  | Phase 2 | 0.740 | 0.825 |
|  | Amplitude 3 | 0.372 | 0.616 |  | Phase 3 | 0.058 | 0.203 |
|  | Amplitude 4 | 0.411 | 0.616 |  | Phase 4 | 0.468 | 0.679 |
|  | Amplitude 5 | 0.584 | 0.757 |  | Phase 5 | 0.455 | 0.679 |
|  | Amplitude 6 | 0.842 | 0.871 |  | Phase 6 | 0.053 | 0.203 |
|  | Amplitude 7 | 0.565 | 0.757 |  | Phase 7 | 0.892 | 0.923 |
|  | Amplitude 8 | 0.634 | 0.757 |  | Phase 8 | 0.927 | 0.927 |
|  | Amplitude 9 | 0.411 | 0.616 |  | Phase 9 | 0.379 | 0.654 |
|  | Amplitude 10 | 0.722 | 0.774 |  | Phase 10 | 0.179 | 0.433 |
| Vertical (z) | Amplitude 1 | **0.009** | 0.092 |  | Phase 1 | 0.720 | 0.825 |
|  | Amplitude 2 | **0.000** | **0.003** |  | Phase 2 | **0.000** | **0.003** |
|  | Amplitude 3 | **0.036** | 0.153 |  | Phase 3 | **0.011** | 0.155 |
|  | Amplitude 4 | 0.230 | 0.576 |  | Phase 4 | **0.034** | 0.203 |
|  | Amplitude 5 | **0.013** | 0.099 |  | Phase 5 | **0.031** | 0.203 |
|  | Amplitude 6 | **0.032** | 0.153 |  | Phase 6 | **0.042** | 0.203 |
|  | Amplitude 7 | 0.274 | 0.616 |  | Phase 7 | 0.383 | 0.654 |
|  | Amplitude 8 | 0.084 | 0.315 |  | Phase 8 | 0.102 | 0.269 |
|  | Amplitude 9 | 0.626 | 0.757 |  | Phase 9 | 0.097 | 0.269 |
|  | Amplitude 10 | 0.681 | 0.757 |  | Phase 10 | 0.271 | 0.604 |

**References**

[S1] T.C. Pataky, Power1D: a Python toolbox for numerical power estimates in experiments involving one-dimensional continua, PeerJ Computer Science. 3 (2017) e125.

[S2] M.A. Robinson, J. Vanrenterghem, T.C. Pataky, Sample size estimation for biomechanical waveforms: Current practice, recommendations and a comparison to discrete power analysis, Journal of Biomechanics. (2021) 110451.

[S3] F. Luciano, L. Ruggiero, G. Pavei, Sample size estimation in locomotion kinematics and electromyography for statistical parametric mapping, Journal of Biomechanics. 122 (2021) 110481.

[S4] A.E. Minetti, C. Cisotti, O.S. Mian, The mathematical description of the body centre of mass 3D path in human and animal locomotion, J Biomech. 44 (2011) 1471–1477.

[S5] K. Takiyama, H. Yokoyama, N. Kaneko, K. Nakazawa, Speed- and mode-dependent modulation of the center of mass trajectory in human gaits as revealed by Lissajous curves, J Biomech. 110 (2020) 109947. https://doi.org/10.1016/j.jbiomech.2020.109947.
